# Prevalence of Elevated Liver Stiffness Among Potential Candidates for Bariatric Surgery in the United States

**DOI:** 10.1007/s11695-021-05885-x

**Published:** 2022-01-05

**Authors:** Stefano Ciardullo, Mattia Pizzi, Pietro Pizzi, Alice Oltolini, Emanuele Muraca, Gianluca Perseghin

**Affiliations:** 1 Department of Medicine and Rehabilitation, Policlinico Di Monza, 20900 Monza, Italy; 2 Department of Medicine and Surgery, University of Milano Bicocca, 20126 Milan, Italy; 3 Centro per lo Studio, la Ricerca e la terapia dell’Obesità, Policlinico Di Monza, 20900 Monza, Italy

**Keywords:** NAFLD, Fibrosis, Obesity, Screening, FibroScan, Cirrhosis

## Abstract

**Purpose:**

Obesity represents a well-known risk factor for metabolic-dysfunction associated fatty liver disease (MAFLD) and its progression towards cirrhosis. The aim of this study is to estimate the proportion of potential candidates to a bariatric surgery intervention that has an elevated liver stiffness on vibration-controlled transient elastography (VCTE).

**Materials and Methods:**

This is a cross-sectional study performed using data obtained during the 2017–2018 cycle of the National Health and Nutrition Examination Survey. Potential candidates for a bariatric surgery intervention from the general US population were identified by applying criteria from international guidelines. All included participants were evaluated by VCTE. A controlled attenuation parameter (CAP) value ≥ 288 dB/m was considered indicative of steatosis while liver stiffness measurement (LSM) was considered elevated if ≥ 9.7 kPa. Multivariable logistic regression models were fitted to identify independent predictors of both outcomes.

**Results:**

A total of 434 participants were included (mean age 42.9 ± 0.6 years; 54.4% women). Among them, 76.7% (95% CI 71.7–81.0) had steatosis, while 23.1% (95% CI 17.8–29.3) had an elevated LSM. Male sex, older age, γ-glutamyltranspeptidase levels, and body mass index (BMI) were independent predictors of steatosis, while BMI was the only independent predictor of elevated LSM. Non-Hispanic black participants were protected from both outcomes, while other ethnicities were not.

**Conclusion:**

The prevalence of elevated LSM is high in potential candidates for a bariatric surgery intervention. Accurate screening for occult advanced liver disease might be indicated in this patient population.

**Graphical abstract:**

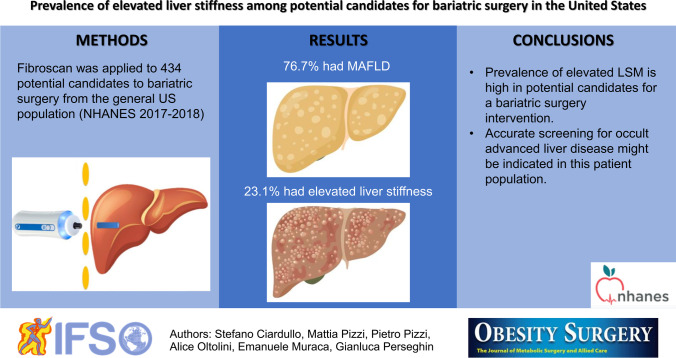

## Introduction

Recent data from the National Center for Health statistics (United States) have shown that the age-adjusted prevalence of obesity in adults is steadily growing, affecting 42.4% of US citizens in 2017–2018 as compared with 22.9% in 1988–1994. In parallel, the prevalence of severe obesity (defined as a body mass index ≥ 40 kg/m^2^) more than tripled in the same period (from 2.8 to 9.2%) [1]. Fueled by this trend, metabolic (dysfunction)-associated fatty liver disease (MAFLD) has increased in prevalence as well, affecting 37–39% of the general adult US population and ~ 75% of patients with type 2 diabetes (T2D) in the most recent studies [2–4]. While most affected patients will not progress towards cirrhosis and decompensation because of competing risks of death from cardiovascular disease and cancer, MAFLD is currently the most rapidly growing indication for liver transplantation, ranking second in the USA [5]. Several studies have now shown that the degree of liver fibrosis represents the best predictor of the future occurrence of clinically relevant liver-related events [6]. In the absence of approved pharmacologic therapy for this condition, lifestyle changes still represent the first-line treatment in clinical practice. In particular, it has been shown that, among patients without advanced fibrosis or cirrhosis at baseline, weight reductions of ≥ 10% can induce a near-universal steato-hepatitis resolution and fibrosis improvement by at least one stage. On the other hand, a seminal study showed that this amount of weight loss could be achieved by only 10% of patients [7]. In this context, bariatric surgery, which is the most effective measure to achieve profound and sustained weight loss, represents a valuable tool for the treatment of MAFLD as well, with large reductions in inflammation and fibrosis following different bariatric procedures [8, 9]. While guidelines do not recommend routine imaging studies to evaluate liver disease in bariatric surgery candidates, they concede that abdominal ultrasonography or elastography may be helpful and may be considered to identify MAFLD and advanced fibrosis and that a comprehensive evaluation (with the possible performance of liver biopsy during the procedure) needs to be performed in patients with suspected cirrhosis [10, 11]. Even though different strategies have been adopted to identify patients at high risk of advanced liver disease, including serum biomarkers and imaging techniques [12, 13], most studies reporting the prevalence of advanced fibrosis were performed in single bariatric surgery units and gave largely variable results [14] and population-based studies are lacking.

Here, we report the prevalence of MAFLD and elevated liver stiffness (as a surrogate of the degree of liver fibrosis) measured by vibration-controlled transient elastography (VCTE) in US adults that meet the criteria for a bariatric surgery intervention. To achieve these goals, we performed a cross-sectional study using data from the most recent cycle of the National Health and Nutrition Evaluation Survey (NHANES), 2017–2018.

## Materials and Methods

This is an analysis of data from the 2017–2018 cycle of NHANES, which is conducted in the United States by the National Center for Health Statistics of the Centers for Disease Control and Prevention. NHANES is a cross-sectional survey program that aims to include an individual representative of the general, non-institutionalized population of all ages. To this end, it recruits approximately 5000 participants per year, applying a stratified, multistage, clustered probability sampling design. In order to provide reliable estimates on minorities, oversampling of non-Hispanic black, Hispanic, and Asian persons, people with low income, and older adults is performed. The survey consists of two main parts: a structured interview conducted in the participants’ home and a standardized health examination conducted in a mobile examination center (MEC). The full methodology of data collection is available elsewhere [15]. The original survey was approved by the Centers for Disease Control and Prevention Research Ethics Review Board and written informed consent was obtained from all adult participants. The present analysis was deemed exempt by the Institutional Review Board at our institution, as the dataset used in the analysis was completely de-identified.

### Laboratory Tests and Clinical Data

Participants self-reported age, sex, ethnicity (categorized as non-Hispanic white, non-Hispanic black, Hispanic, or other), education, smoking status, and previous medical history. Body measurements including height (cm), weight (kg), and waist circumference (cm) were ascertained during the mobile examination center visit; body mass index (BMI) was calculated as weight in kilograms divided by height in meters squared. Hypertension was defined as systolic blood pressure (SBP) value ≥ 140 mmHg and/or a diastolic blood pressure (DBP) value ≥ 90 mmHg or currently taking antihypertensive drugs [16]. Diabetes was defined in accordance with the American Diabetes Association criteria if any of the following conditions were met: (1) a self-reported diagnosis of diabetes; (2) use of anti-diabetic drugs; (3) a hemoglobin A1c (HbA1c) level ≥ 6.5% (48 mmol/mol); (4) a fasting plasma glucose ≥ 126 mg/dl; (5) a random plasma glucose ≥ 200 mg/dl [17].

Laboratory methods for measurements of HbA1c, glucose, lipid profile, alanine aminotransferase (ALT), aspartate aminotransferase (AST), γ-glutamyltranspeptidase (GGT), platelet count, creatinine, and albumin are reported in detail elsewhere (18). Hepatitis C virus infection was indicated by the presence of viral RNA and/or a confirmed antibody test and hepatitis B virus infection as a positive surface antigen test, as described [19]. The fibrosis-4 (FIB-4) index, which is based on AST, ALT, platelet count, and age, was calculated as originally described [20]. A cut-off of 1.3 was used to exclude the presence of advanced liver fibrosis [21].

Estimated glomerular filtration rate (eGFR) was computed according to the Chronic Kidney Disease Epidemiology Collaboration (CKD-EPI) equation and CKD was defined as an eGFR < 60 ml/min/1.73 m^2^. Urine albumin to creatinine ratio (UACR) was considered elevated if ≥ 30 mg/g. Information regarding smoking status and history of cardiovascular disease (CVD) was based on self-report.

### Vibration-Controlled Transient Elastography

In the 2017–2018 cycle, VCTE was performed by NHANES technicians after a 2-day training program with an expert technician, using the FibroScan® model 502 V2 Touch (Echosens, Paris, France) equipped with a medium (M) and extra-large (XL) probes. The M probe was used initially unless the machine indicated the use of the XL probe. Inter-rater reliability between health technicians and expert FibroScan® technicians (tested on 32 subjects) was 0.86 for stiffness (mean difference 0.44 ± 1.3 kPa) and 0.94 for CAP (mean difference 4.5 ± 19.8 db/m).

Exams were considered reliable only if at least 10 liver stiffness measurements (LSM) were obtained after a fasting time of at least 3 h, with an interquartile (IQRe) range/median < 30%. Median controlled attenuation parameter (CAP) values ≥ 288 dB/m were considered indicative of any degree of liver steatosis in accordance with a recent study by Caussy et al. [22]. In the main analysis, a median LSM value ≥ 9.7 kPa was considered elevated, as it represented the Youden-index derived cut-off for identifying advanced fibrosis (≥ F3) in a recent study by Eddowes et al. [23]. We also applied thresholds of 14.1 kPa and 20.9 kPa, which were associated with 90% specificity for advanced fibrosis and cirrhosis, respectively, in the same study using liver biopsy as the gold standard.

### Analysis Sample

Based on the criteria developed by the National Institutes of Health (NIH) Consensus Development Panel in 1991 [24] and reviewed by the American Association of Clinical Endocrinologists, the Obesity Society, and American Society for Metabolic & Bariatric Surgery in 2013 [25], participants with the following features were considered potential candidates for a bariatric surgical procedure:

- 18–60 years of age
- BMI > 40 kg/m^2^ independently of chronic complications
- BMI ≥ 35 kg/m^2^ in the presence of chronic complications

For the current study, as data on some complications included in the guidelines were not available, only the following were considered:

- Hypertension (either known or discovered during the survey)
- Type 2 diabetes (either known or discovered during the survey)
- Dyslipidemia (based on the current use of lipid-lowering medications)

**Fig. 1:**
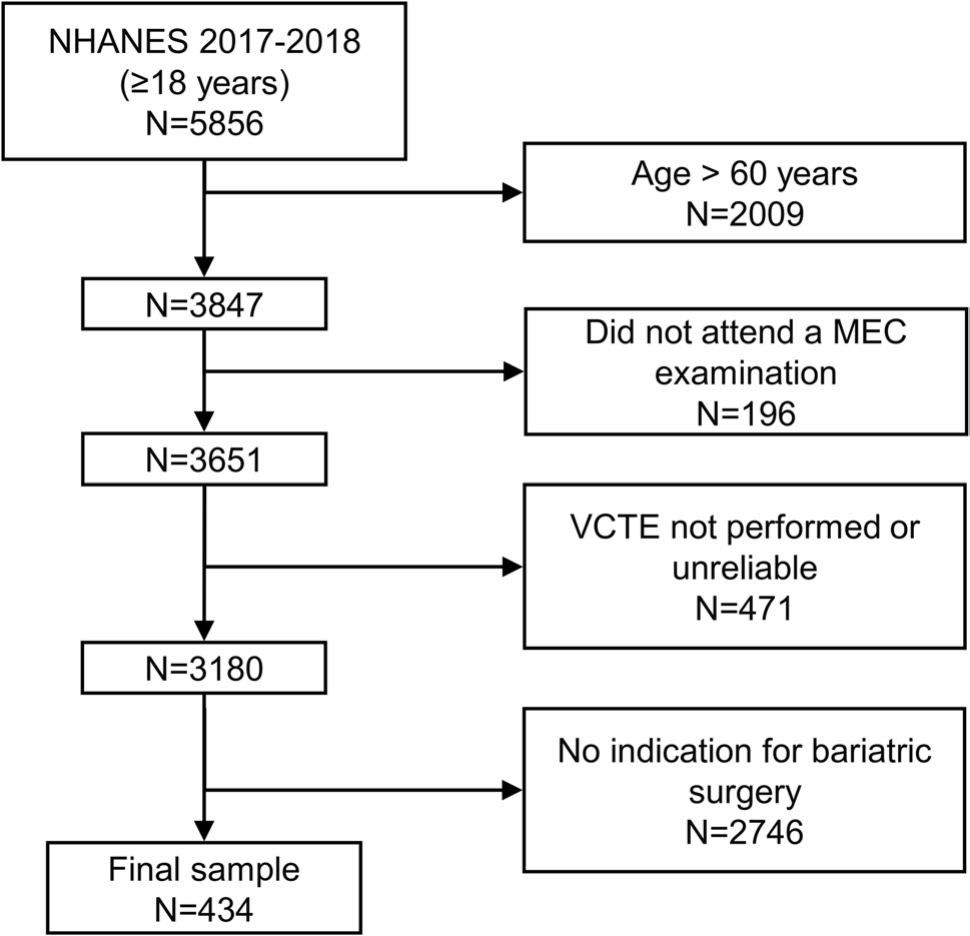
Flowchart of the study participants. Abbreviations: NHANES, National Health and Nutrition Examination Survey

From a total of 5856 adult participants included in the survey, 3847 were aged 18–60 and, of those, 3651 attended a MEC visit. We initially excluded individuals without a reliable VCTE exam, leading to 3180 potential candidates. Finally, by applying inclusion criteria for a bariatric surgery intervention, our final sample consisted of 434 individuals (Fig. 1).

### Statistical Analysis

All analyses were conducted using Stata version 16 (StataCorp, College Station, TX), accounting for the complex survey design of NHANES. We used appropriate weighting for each analysis, as suggested by the NCHS. Data are expressed as weighted proportions for categorical variables and as weighted means and the corresponding standard error (SE) for continuous variables.

Participants’ characteristics by liver steatosis and fibrosis status were compared using linear regression for continuous variables and the design-adjusted Rao-Scott chi-square test for categorical variables. Logistic regression analysis was performed in order to evaluate the effect of different variables on the presence of steatosis and fibrosis. A biological plausibility approach was followed for the choice of predictors including known risk factors for steatosis such as age, sex, ethnicity, diabetes, BMI, and liver enzymes. Cohen’s κ was run to determine the degree of agreement between FIB-4 and VCTE in identifying advanced liver fibrosis. A two-tailed value of *p* < 0.05 was considered statistically significant.

## Results

Of the 434 patients with reliable elastographic exams, 68 (15.7%) were investigated using the M probe and 366 (84.3%) using the XL probe. The mean age was 43.3 years, the mean BMI was 42.5 kg/m^2^, and 54.4% were women.

### Prevalence of Steatosis and Elevated LSM

The distribution of the study population according to the presence of MAFLD is shown in Table 1. The weighted prevalence of steatosis was 76.7% (95% CI 71.7–81.0), which was significantly higher than the prevalence of steatosis identified in NHANES individuals in the same age group that did not meet bariatric surgery criteria (24.4, 95% CI 20.5–28.8, *p* < 0.001).

**Table 1:** Features of the study population according to controlled attenuation parameter (CAP) values

	Entire cohort	CAP < 288 dB/m	CAP ≥ 288 dB/m	*p* value
Age (years)	43.3 (0.6)	38.4 (1.2)	44.8 (0.6)	< 0.001
Female (%)	54.4 (3.5)	79.3 (7.6)	49.1 (3.9)	0.011
Cigarette smoke (%)				0.948
Never	55.7 (3.3)	55.1 (5.8)	55.8 (3.3)	
Former	27.6 (3.5)	28.3 (5.0)	27.4 (4.2)	
Current	16.7 (2.7)	16.6 (3.7)	16.8 (2.6)	
Ethnicity (%)				0.024
Non-Hispanic white	58.0 (3.8)	47.5 (7.6)	61.2 (4.0)	
Hispanic	16.0 (2.2)	10.8 (4.1)	17.6 (2.5)	
Non-Hispanic black	17.6 (3.4)	30.6 (6.4)	13.7 (2.7)	
Non-Hispanic Asian	1.3 (0.4)	1.5 (0.9)	1.2 (0.5)	
Other	7.1 (2.0)	9.7 (2.1)	6.3 (2.6)	
BMI (kg/m^2^)	42.5 (0.4)	42.1 (0.9)	42.6 (0.4)	0.528
HbA1c (%)	6.1 (0.1)	5.8 (0.1)	6.3 (0.1)	0.014
AST (IU/L)	21.9 (0.5)	18.9 (1.3)	22.8 (0.9)	0.064
ALT (IU/L)	27.8 (0.8)	18.8 (1.7)	30.5 (1.4)	< 0.001
GGT (IU/L)	37.3 (1.1)	24.9 (2.7)	41.2 (1.6)	< 0.001
Albumin (g/dL)	3.9 (0.0)	3.9 (0.1)	3.9 (0.0)	0.428
Platelet count (10^9^/L)	272.3 (5.5)	293.9 (9.8)	265.6 (6.7)	0.030
Total cholesterol (mg/dL)	186.8 (3.7)	182.0 (6.0)	188.3 (3.7)	0.287
Triglycerides (mg/dL)	170.3 (9.0)	127.7 (9.6)	183.5 (10.7)	< 0.001
HDL-cholesterol (mg/dL)	45.6 (0.6)	49.5 (1.3)	44.5 (0.8)	0.006
Type 2 diabetes (%)	27.5 (3.2)	14.1 (3.8)	31.6 (4.0)	0.007
Hypertension (%)	53.5 (4.4)	49.9 (8.7)	54.6 (4.3)	0.621
CKD (%)	2.2 (1.0)	0.2 (0.2)	2.8 (1.2)	0.003
CVD (%)	6.2 (1.1)	5.3 (2.7)	6.5 (1.1)	0.711
UACR (mg/g, %)				0.167
< 30	83.1 (2.8)	88.9 (3.0)	81.4 (3.6)	
30–300	13.3 (2.6)	9.8 (2.9)	14.4 (3.3)	
> 300	3.5 (1.3)	1.2 (0.3)	4.2 (1.7)	

Data are expressed as weighted proportions (± standard error (SE)) for categorical variables and as weighted means ± SE for continuous variables. Linear regression and Rao-Scott chi-square test were used to compare groups

*BMI*, body mass index; *UACR*, urinary albumin creatinine ratio; *HbA1c*, hemoglobin A1c; *AST*, aspartate aminotransferase; *ALT*, alanine aminotransferase; *GGT*, gamma-glutamyltranspeptidase; *HDL*, high-density lipoprotein

Participants with MAFLD were significantly older, more commonly men of non-Hispanic white and Hispanic ethnicity, and less frequently non-Hispanic black. They showed higher HbA1c, ALT, GGT, and triglycerides levels and lower HDL cholesterol and platelet count. No difference was found in BMI, cigarette smoke, and albumin. In terms of comorbidities, they showed a significantly higher prevalence of T2D and reduced eGFR, with no significant differences in hypertension and increased UACR.

As shown in Table 2, the weighted prevalence of elevated LSM (≥ 9.7 kPa) was 23.1% (95% CI 17.8–29.3). Also, in this case, the prevalence was significantly higher than in NHANES participants that did not meet the criteria for bariatric surgery (1.8%, 95% CI 1.1–2.8, *p* < 0.001). Patients with elevated LSM had a higher BMI, ALT, and AST levels, with no significant differences in age, sex, cigarette smoke, the proportion of patients with T2D, CVD, CKD, and hypertension.

**Table 2:** Features of the study population according to liver stiffness measurement (LSM) values

	LSM < 9.7	LSM > 9.7	*p* value
Age (years)	42.7 (0.8)	45.0 (1.6)	0.232
Female (%)	58.1 (4.3)	42.1 (8.1)	0.132
Ethnicity (%)			0.219
Non-Hispanic white	56.1 (4.2)	64.4 (8.3)	
Hispanic	16.9 (2.5)	13.2 (3.4)	
Non-Hispanic black	19.6 (3.9)	11.0 (3.1)	
Non-Hispanic Asian	1.0 (0.3)	2.3 (1.2)	
Other	6.5 (1.5)	9.2 (4.8)	
Cigarette smoke (%)			0.097
Never	52.2 (3.5)	67.3 (6.3)	
Former	29.8 (3.7)	20.4 (5.6)	
Current	18.0 (3.1)	12.3 (3.0)	
BMI (kg/m^2^)	41.7 (0.4)	45.2 (1.0)	0.002
HbA1c (%)	6.1 (0.1)	6.2 (0.2)	0.787
AST (IU/L)	20.5 (0.5)	26.6 (1.8)	0.005
ALT (IU/L)	25.8 (1.0)	34.2 (2.1)	0.003
GGT (IU/L)	34.6 (1.6)	46.6 (5.3)	0.083
Albumin (g/dL)	3.9 (0.0)	4.0 (0.0)	0.088
Platelet count (109/L)	276.1 (6.5)	259.9 (9.6)	0.159
Total cholesterol (mg/dL)	186.3 (3.4)	188.4 (7.4)	0.747
Triglycerides (mg/dL)	170.5 (7.5)	169.4 (22.8)	0.957
HDL-cholesterol (mg/dL)	46.3 (0.8)	43.5 (1.6)	0.171
Type 2 diabetes (%)	25.9 (3.3)	32.9 (10.0)	0.508
Hypertension (%)	54.5 (4.5)	50.1 (8.2)	0.487
CKD (%)	2.1 (1.2)	2.2 (1.3)	0.976
CVD (%)	6.6 (1.5)	5.0 (1.3)	0.480

Data are expressed as weighted proportions (± standard error (SE)) for categorical variables and as weighted means ± SE for continuous variables. Linear regression and Rao-Scott chi-square test were used to compare groups

*BMI*, body mass index; *UACR*, urinary albumin creatinine ratio; *HbA1c*, hemoglobin A1c; *AST*, aspartate aminotransferase; *ALT*, alanine aminotransferase; *GGT*, gamma-glutamyltranspeptidase; *HDL*, high-density lipoprotein

As sensitivity analyses, we used LSM cut-offs with 90% specificity derived from Eddowes et al. [23] (Table 3). Suspected significant fibrosis, advanced fibrosis, and cirrhosis were present in 13.8% (95% CI 10.3–18.2%), 11.2% (95% CI 7.7–15.9%), and 6.2% (95% CI 4.6–8.2%) of patients with thresholds of 12.1 kPa, 14.1 kPa, and 20.9 kPa, respectively.

**Table 3:** Prevalence of suspected significant fibrosis, advanced fibrosis, and cirrhosis in the studied population

LSM cut-off (kPa)	Prevalence (%)
Significant fibrosis (≥ F2)	
8.2 (Youden index)	30.4 (24.3–37.2)
12.1 (90% Sp)	13.8 (10.3–18.2)
Advanced fibrosis (≥ F3)	
9.7 (Youden index)	23.1 (17.8–29.3)
14.1 (90% Sp)	11.2 (7.7–15.9)
Cirrhosis (F4)	
13.6 (Youden index)	12.0 (8.4–16.9)
20.9 (90% Sp)	6.2 (4.6–8.2)

Cut-offs are derived from the study of Eddowes et al. [23]. *LSM*, liver stiffness measurement

### Independent Predictors of Steatosis and Fibrosis

On multivariable logistic regression analysis age, BMI, and GGT values were positively associated with steatosis, whereas non-Hispanic black ethnicity and female sex were associated with lower odds. No differences were found for other ethnicities (Table 4). Moreover, higher BMI was the only variable that was positively associated with elevated LSM. Similar to steatosis, non-Hispanic black patients had a significantly lower risk.

**Table 4:** Multivariable logistic regression model assessing the contribution of several predictors on the odds of increased Controlled Attenuation Parameter (CAP) and Liver Stiffness Measurement (LSM) in the studied population

	CAP ≥ 288 dB/m			LSM ≥ 9.7 kPa		
	OR	95% CI	*p* value	OR	95% CI	*p* value
Age (years)	1.06	1.03–1.09	< 0.01	1.03	0.99–1.08	0.15
Female sex	0.35	0.12–1.01	0.05	0.48	0.18–1.28	0.13
Ethnicity						
Non-Hispanic white	1.0			1.0		
Hispanic	2.28	0.61–8.52	0.20	0.83	0.29–2.38	0.71
Non-Hispanic black	0.38	0.21–0.70	< 0.01	0.39	0.16–0.94	0.04
Non-Hispanic Asian	0.65	0.08–5.13	0.66	2.06	0.54–7.95	0.27
Other	0.55	0.16–1.91	0.32	1.09	0.34–3.55	0.87
BMI (kg/m^2^)	1.09	1.01–1.18	0.03	1.14	1.07–1.21	< 0.01
Type 2 diabetes	2.16	0.88–5.35	0.09	1.30	0.33–5.23	0.69
GGT (IU/L)	1.03	1.00–1.07	0.05	1.01	1.00–1.03	0.16

*OR*, odds ratio; *BMI*, body mass index; *ALT*, alanine aminotransferase; *AST*, aspartate aminotransferase; *GGT*, gamma-glutamyltransferase; *DM*, diabetes mellitus

In the entire population, advanced liver fibrosis could not be excluded in 32 participants (weighted prevalence 6.3%) when a FIB-4 cut-off of 1.3 was applied. No significant correlation was found between FIB-4 and LSM when considered as continuous variables (*r* = 0.07, *p* = 0.165). Agreement between FIB-4 and LSM was low even in categorical analysis using cut-offs of 1.3 and 9.7 kPa, respectively (*κ* = 0.08, *p* = 0.025).

## Discussion

In the present study, we report the prevalence of MAFLD and elevated LSM assessed by VCTE in a nationally representative sample of potential candidates to a bariatric surgery intervention from the US. By applying validated cut-offs for both CAP and LSM values, we estimate that 76.7% of patients have evidence of MAFLD, 23.1% have LSM values indicative of advanced (≥ F3) fibrosis, and 6.2% have values indicative of cirrhosis. These prevalence rates were significantly higher compared with those of NHANES individuals in the same age group that did not meet bariatric surgery criteria. While we identified several predictors of MAFLD (including age, sex, ethnicity, and liver enzymes values), BMI and ethnicity were the only independent predictors of elevated LSM in the studied population.

There is an intense debate in the scientific community on whether systematic screening for MAFLD and associated fibrosis should be performed in at-risk populations, and, if so, how. While the European Association for the Study of the Liver (EASL), Diabetes (EASD), and Obesity (EASO) guidelines recommend screening for MAFLD in patients with obesity and in patients with T2D [26], the American Association for the Study of Liver Diseases (AASLD) does not recommend routine screening but only suggests case finding [27]. Evidence on the cost-effectiveness of different screening approaches is lacking and no pharmacological treatment is available; nonetheless, identification of patients with advanced fibrosis might be instrumental for identifying compensated cirrhosis, initiating hepatocellular carcinoma surveillance or enrolling the patient in a randomized clinical trial [28]. Currently, data on the prevalence of MAFLD and advanced liver fibrosis in the setting of bariatric surgery candidates come from case series performed in specialized centers in which liver biopsy was performed at the time of surgery. In a meta-analysis including 12 studies with available histologic data, Machado et al. reported a prevalence of steatosis of 91% (range: 85–98%), whereas 10% (range: 4–16%) and 1.7% (range: 1–7%) had advanced fibrosis and occult cirrhosis, respectively; bariatric surgery clinics might therefore be considered a potential setting to identify and manage these liver conditions.

Routine performance of liver biopsy is not indicated by recent guidelines [11], given that the majority of patients have simple steatosis or a low degree of fibrosis and the possible bleeding risks associated with the procedure. In the present study, we show that by applying VCTE to a large and representative sample of US adults who qualify for bariatric surgery, approximately 25% have elevated LSM values. Given the high negative predictive value of VCTE, routine performance of this exam would limit the number of liver biopsies to be performed by focusing on individuals at higher risk.

Knowing that the patient has advanced fibrosis or cirrhosis has a series of important implications. Careful evaluation of portal hypertension should be performed in patients with cirrhosis prior to surgery as a decompensated disease is considered a contraindication to the procedure [10]. These patients are also at higher bleeding risk, leading surgeons to particular attention to hemostasis. Surveillance for hepatocellular carcinoma is indicated in the presence of biopsy-proven cirrhosis or in the case of elevated non-invasive markers (FIB-4 > 2.67 and/or LSM > 16.1 kPa) [28]. The identification of patients without cirrhosis, but with significant/advanced fibrosis (F2-F3), is still important and endorsed by the European guidelines [26], as these individuals are at higher risk of liver-related events in the subsequent years and might be followed more closely in a hepatologic setting or included in a clinical trial.

Moreover, VCTE might be used to evaluate the impact of bariatric surgery on liver disease. A recent study including 76 patients who underwent liver biopsy at the time of bariatric surgery and at 1-year follow-up showed that significant improvement occurred on liver histology in terms of inflammation, ballooning, and fibrosis. Interestingly, these changes were paralleled by similar improvements in both CAP and LSM [29]. Considering that VCTE is a fast and well-tolerated non-invasive exam that can be performed by trained non-medical personnel, even if cost-effectiveness studies are still lacking on its widespread use in clinical practice, we believe that its application in the setting of bariatric surgery might be the first step not to miss those patients that might develop cirrhosis in the following decades.

Our study has the strength of focusing on a large sample of unselected patients from the general US population rather than on selected patients being evaluated in a single bariatric surgery unit. This allows us to provide estimates that are representative of all patients with severe obesity from the USA. Moreover, we used one of the best-performing non-invasive tests for non-invasively assessing liver steatosis and fibrosis. In this sense, the availability of the XL probe, which was essential in our population, made it possible to obtain valid LSM measurements in the vast majority of patients. On the same lines, we acknowledge the presence of some limitations. First, the absence of histologic data prevents us from reporting the exact prevalence of steatosis and advanced fibrosis according to the gold standard technique. On the other hand, performing liver biopsy in participants from the general population was not indicated and might cause unnecessary harm [30]. Previous studies showed that VCTE has good accuracy also in the setting of individuals with severe obesity when compared with intra-operative liver biopsy, with areas under the receiver operator characteristic curve ranging from 0.83 to 0.87 to identify advanced (≥ F3) fibrosis [29, 31, 32]. Nonetheless, positive predictive values were relatively low [33], suggesting that its performance is higher in excluding rather than confirming the presence of advance fibrosis. In this context, our estimates should not be strictly interpreted as direct estimates of advanced liver fibrosis or cirrhosis, but rather of the proportion of patients at increased risk for these histologic changes, in which intraoperative liver biopsy might be indicated and stricter follow-up be planned accordingly.

Second, there is no universal cut-off guideline for CAP score [23]. However, we employed the one proposed by Caussy et al., which was derived from a US population using magnetic resonance imaging–proton density fat fraction as a reference gold standard technique [34, 35].

In conclusion, US adults with severe obesity that may benefit from a bariatric surgery intervention have a high prevalence of both steatosis and elevated LSM. The use of VCTE in the pre-surgical assessment might identify the subset of patients (~ 25%) that should be more thoroughly evaluated from a liver-related standpoint.

## Data Availability

All data used in this study are publicly available online at the NHANES website.

## Code Availability

Code is available from the corresponding author upon reasonable request.
